# The Exposure–Response Curve for Ozone and Risk of Mortality and the Adequacy of Current Ozone Regulations

**DOI:** 10.1289/ehp.8816

**Published:** 2006-01-23

**Authors:** Michelle L. Bell, Roger D. Peng, Francesca Dominici

**Affiliations:** 1 School of Forestry and Environmental Studies, Yale University, New Haven, Connecticut, USA; 2 Department of Biostatistics, Johns Hopkins Bloomberg School of Public Health, Baltimore, Maryland, USA

**Keywords:** mortality, ozone, regulations, threshold

## Abstract

Time-series analyses have shown that ozone is associated with increased risk of premature mortality, but little is known about how O_3_ affects health at low concentrations. A critical scientific and policy question is whether a threshold level exists below which O_3_ does not adversely affect mortality. We developed and applied several statistical models to data on air pollution, weather, and mortality for 98 U.S. urban communities for the period 1987–2000 to estimate the exposure–response curve for tropospheric O_3_ and risk of mortality and to evaluate whether a “safe” threshold level exists. Methods included a linear approach and subset, threshold, and spline models. All results indicate that any threshold would exist at very low concentrations, far below current U.S. and international regulations and nearing background levels. For example, under a scenario in which the U.S. Environmental Protection Agency’s 8-hr regulation is met every day in each community, there was still a 0.30% increase in mortality per 10-ppb increase in the average of the same and previous days’ O_3_ levels (95% posterior interval, 0.15–0.45%). Our findings indicate that even low levels of tropospheric O_3_ are associated with increased risk of premature mortality. Interventions to further reduce O_3_ pollution would benefit public health, even in regions that meet current regulatory standards and guidelines.

Tropospheric ozone is a common urban area pollutant linked to numerous harmful health effects, including reduced lung function, increased frequency of respiratory symptoms, and development of asthma [Broeckaert et al. 1999; Brunekreef and Holgate 2002; McConnell et al. 2002; U.S. Environmental Protection Agency (EPA) 1996]. Recent meta-analysis and time-series studies have linked short-term O_3_ exposure to premature mortality (Anderson et al. 2004; Bell et al. 2004, 2005; Huang et al. 2005; Ito et al. 2005; Levy et al. 2005), but the exposure–response curve for O_3_ remains unknown. More than 100 million people in the United States live in areas that exceed the current health-based U.S. National Ambient Air Quality Standard (NAAQS) for O_3_ (U.S. EPA 2004). Elevated concentrations of O_3_ are also a growing concern for rapidly developing nations with rising emissions of O_3_ precursors from expanding transportation networks.

The U.S. EPA is currently reviewing the scientific evidence on O_3_ and health to determine if the current NAAQS (80 ppb for the daily 8-hr maximum) should be revised to meet the goal mandated in the Clean Air Act Amendments (1990) to protect human health with an “adequate margin of safety” (U.S. EPA 1997). There are several critical questions regarding the association between O_3_ and mortality as the current NAAQS is re-examined: Can O_3_ affect mortality even at low levels? Are current regulations sufficiently stringent to prevent premature mortality? Is there an attainable threshold O_3_ level that does not affect mortality, and if so, is it below current regulatory limits? Evidence relevant to these questions can be obtained by estimating the exposure–response curve for O_3_ and mortality. The shape of this curve can provide a basis for *a*) understanding the impacts of low levels of O_3_ pollution on health, *b*) assessing the adequacy of regulatory standards, *c*) designing other health-based studies on O_3_, *d*) estimating the health consequences associated with emissions scenarios and policies (e.g., Hubbell et al. 2005), and *e*) assessing how climate change might affect human health through altered O_3_ levels (e.g., Knowlton et al. 2004).

## Materials and Methods

### Data and hierarchical model.

**Figure 1:**
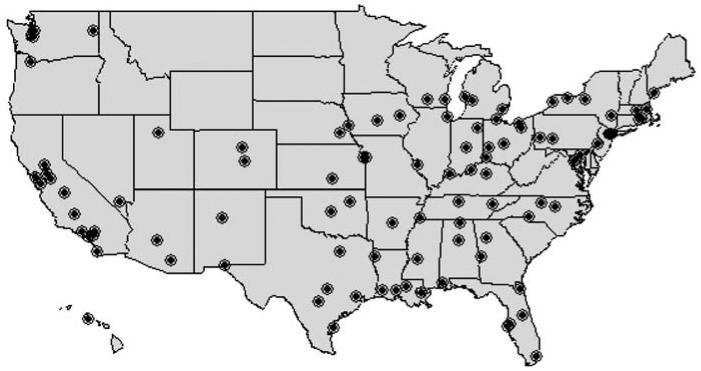
Locations of the 98 U.S. urban communities examined in this study.

To investigate the exposure–response relationship between O_3_ and mortality, we applied several modeling structures to daily time-series data on all-cause nonaccidental mortality, weather (temperature and dew point), and O_3_ pollution levels for the period 1987–2000 for 98 large U.S. urban communities (Figure 1). The communities are listed in the Appendix and consist of urban areas based on a county or a set of contiguous counties. Our database includes > 40% of the total U.S. population and is part of the National Morbidity, Mortality, and Air Pollution Study (NMMAPS) (Daniels et al. 2000, 2004; Dominici et al. 2000; Samet et al. 2000a, 2000b, 2000c). We obtained air pollution data by request from the U.S. EPA, and weather data from the U.S. National Climatic Data Center.

We used measurements from ambient monitors as a surrogate for community-level exposure. The measure of exposure was the average of the same and previous days’ O_3_ levels (lag 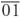). First, 24-hr averages were calculated for each day within each community, and then the lag 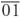 concentrations were calculated. The use of any single day’s O_3_ level as the exposure metric would underestimate the relationship between O_3_ and mortality (Bell et al. 2004). We aggregated measurements from multiple monitors within a community using a 10% trimmed mean to estimate a community-level exposure.

We obtained mortality data by request from the National Center for Health Statistics. The mortality outcome is the number of daily deaths within the community excluding nonresidents and excluding those caused by injuries and other external causes corresponding to *International Classification of Diseases, 9th Revision* (ICD-9) [World Health Organization (WHO) 1978], codes 800 and above, and *International Classification of Diseases, 10th Revision* (ICD-10) (WHO 1993), codes S and above. Additional information on the generation of the air pollution data set and the entire database is available through the Internet-Based Health & Air Pollution Surveillance System (iHAPSS) (iHAPSS 2006).

We used a Bayesian hierarchical model to evaluate the relationship between ambient O_3_ levels and mortality rates within each community (community-specific relative rate estimate) and to combine information across communities to produce a national average relative rate estimate, accounting for the uncertainty of each community’s relative rate (Dominici et al. 2000; Everson and Morris 2000). The first stage estimates the relationship between short-term exposure to O_3_ and daily nonaccidental mortality rates within each community, using a Poisson regression model (McCullagh and Nelder 1989) of the form:


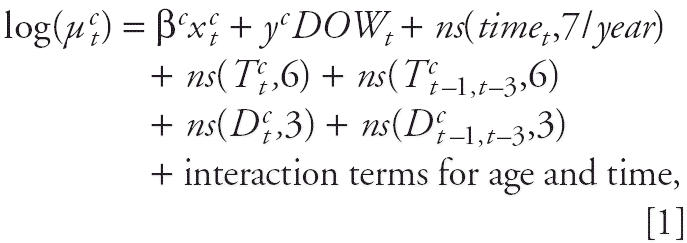


where μ*_t_^c^* is the expected number of deaths for community *c* on day *t*, based on an over-dispersed Poisson distribution; *x_t_^c^* is the average of the same and previous days’ daily O_3_ concentrations in community *c* on day *t*; DOW*_t_* is the categorical variable for day of the week on day *t; ns*(*time_t_* ,7/year) is the natural cubic spline function of calendar time with 7 degrees of freedom per year; *ns*(*T_t_^c^* *,*6) is the natural cubic spline function for temperature with 6 degrees of freedom; *ns*(*T^c^_t_*_−1,_*_t_*_−3_,6) is the natural cubic spline function of the average of the 3 previous days’ temperature (adjusted for current day temperature); *ns*(*D_t_^c^,*3) is the natural cubic spline function for dew point with 3 degrees of freedom; and *ns*(*D^c^_t_*_−1,_*_t_*_−3_,3) is the natural cubic spline function of the average of the 3 previous days’ dew point (adjusted for current day dew point). Interaction terms for age and time are the interaction terms between natural cubic spline functions of time- and age-specific indicators (< 65, 65–74, and ≥ 75 years).

In the first stage, we estimated the effect of O_3_ on mortality for each community, 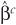, (an estimate of the true community-specific relative rate, β*^c^*), and the corresponding variance 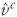. We assume:


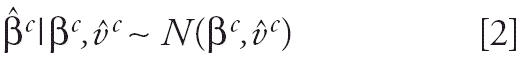



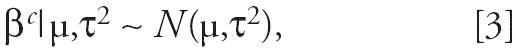


where μ is the true national average relative rate and τ^2^ is the variance of the true community-specific relative rates, β*^c^*. Sensitivity analyses and characteristics of the first-stage statistical model for confounding adjustment have been explored for particulate matter (PM), with results indicating that national average estimates are robust to model specification for weather and seasonal confounding (Peng et al. 2005; Welty and Zeger 2005). Earlier analysis showed national-average and community-specific estimates for O_3_ and mortality to be robust to inclusion of PM_10_ (PM with an aerodynamic diameter < 10 μm) in the first-stage model (Bell et al. 2004). Results were also robust to exclusion of days with high temperature (Bell et al. 2004).

As a second stage, we generated a national relative rate estimate that accounts for the statistical uncertainty of each community’s relative rate estimate and for the variability across communities of the true relative rates. We fit this two-stage normal–normal model by use of a two-level normal independent sampling estimation (TLNise 2006) with noninformative priors (Everson and Morris 2000).

Using this two-stage approach, we performed four analyses that make different modeling assumptions about the community-specific exposure–response curve for O_3_ and mortality. Under each analysis, we estimated a national relative rate and/or a national exposure–response curve by combining information across the 98 communities.

### Linear approach.

For the first analysis, the linear approach, we estimated a linear association between the log of the expected mortality rate and O_3_ levels as described in Equation 1. This model assumes that any change in O_3_ concentration, even at very low levels, can be associated with mortality. For example, a 10-ppb increase in O_3_ levels from 5 to 15 ppb would lead to the same percentage increase in mortality as a 10-ppb rise from 50 to 60 ppb. This is the modeling approach used in most epidemiologic studies of air pollution and in most health and impact assessments of air pollution policies. We then relaxed this assumption of linearity across the entire range of O_3_ levels with the three approaches described below.

### Subset approach.

Under the second analysis, the subset approach, we estimated a linear relationship between the log of the expected mortality rate and O_3_ levels as in Equation 1 but using a subset of the data including only days with lag 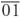 O_3_ levels below a specified concentration, *s*. We performed this analysis for values of *s* ranging from 5 to 60 ppb. Under this approach, we assume that “safe” O_3_ levels are those lower than the specific *s* value that leads to lack of evidence of an association between O_3_ and mortality.

We also used the subset approach to assess the relationship between O_3_ and mortality under several idealized policy scenarios in which various O_3_ regulations and guidelines were met every day in each community. Because O_3_ regulations are expressed in different metrics, we proceeded in three steps. First, we used hourly O_3_ concentrations to calculate daily O_3_ levels under the same metric specified by the standard (e.g., daily 8-hr maximum or daily 1-hr maximum). Second, we constructed a subset of the data set that includes only days that meet the regulatory standard or guideline. For example, for the U.S. EPA O_3_ standard, we first calculated a daily time series of 8-hr maximum O_3_ levels, and then we constructed a subset of the data set that only includes days with an 8-hr maximum O_3_ level < 84 ppb (U.S. EPA 1997). Third, using only days that met the standard, we estimated the percentage increase in mortality associated with a 10-ppb increase in lag 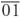 O_3_ levels on average across the 98 communities, with the 95% posterior interval, which is the Bayesian analogue of the 95% confidence interval. This strategy allows us to analyze the subset of days that meet a regulatory requirement using the metric specified in the standard but to present results with a single metric for the exposure variable (lag 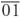 of the 24-hr averages) to maintain a common interpretation of the relative rate estimates.

The NAAQS for O_3_ is “80 ppb” for the daily 8-hr maximum, but U.S. EPA regulations specify that values between 80 and 84 ppb can be rounded down and are not considered exceedances (U.S. EPA 1997). Thus, for our analysis of the NAAQS, we considered a standard of 84 ppb for the daily 8-hr maximum. Regulations generally do not require every monitor to meet the standard every day. For example, a standard can allow a specified number of exceedances and require that a certain percentile (e.g., 98th) meet the requirement on a 3-year average. In actual compliance with a regulatory standard for a given area, the levels of pollution would follow an uneven spatial distribution (U.S. EPA 2005). Our analysis considers a more stringent application in that it incorporates only days with O_3_ levels at or below the specific standard for both the same and previous days. However, the regulatory standard requires compliance from every monitor, whereas this analysis considers averages across communities rather than individual monitor exceedances.

### Threshold approach.

If a threshold (*h*) exists, we would expect to detect an association between O_3_ and mortality for O_3_ levels > *h* but not for O_3_ levels < *h*. Our threshold approach has the same structure of Equation 1, but with the pollution term replaced by


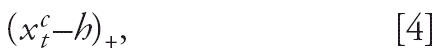


where


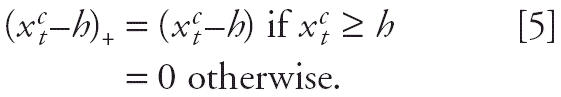


Under this model, we assume no association between O_3_ and mortality for days with O_3_ concentrations below *h* and a linear relationship for days with O_3_ levels above *h*. We performed this analysis for values of *h* ranging from 0 to 60 ppb at increments of 5 ppb. For each community-specific model and threshold level (*h*), we calculated the Akaike Information Criterion (AIC) (Akaike 1973) as


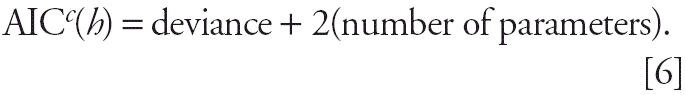


Note that the number of parameters can differ by urban community because of the varying frequencies with which O_3_ is measured and the variables for time. We then calculated the average AIC for each *h* value as


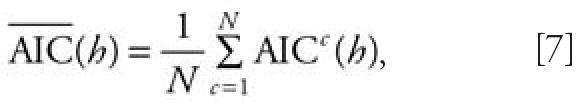


where *n* = number of communities (98). The rationale for this approach is that if an O_3_ threshold exists, the threshold approach with the appropriate value for *h* will have the best fit and therefore the minimum 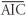 (*h*) (Akaike 1973).

### Spline approach.

Under the fourth analysis, the spline approach, we allow the relationship between O_3_ and mortality to fluctuate for different ranges of pollution levels, using a nonlinear function of O_3_. This model can be defined as Equation 1 but replacing β*^c^x_t_^c^* with *ns*(*x_t_^c^*), where *ns* is a natural cubic spline of O_3_ levels (Daniels et al. 2000, 2004; Dominici et al. 2002). Boundary knots were specified at 0 and 80 ppb, with interior knots at 20 and 40 ppb. The spline approach extends the linear approach because here the relative rate corresponding to a 10-ppb increase in O_3_ levels from 5 to 15 ppb is allowed to differ from the relative rate corresponding to a 10-ppb increase from 50 to 60 ppb. Visual inspection of the estimated exposure–response curve can provide evidence about whether a safe level exists and at what concentration.

## Results

We found that daily increases in ambient O_3_ levels were significantly associated with daily increases in the number of deaths, on average, across the 98 U.S. communities. Specifically, under the linear approach, we found that the percentage increase in all-cause mortality associated with a 10-ppb increase in the lag 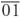 O_3_ levels was 0.32% (95% posterior interval, 0.17–0.46%). We also found that the largest relative rate estimates occur on more recent days: the percentage increases (95% posterior intervals) in all-cause mortality associated with a 10-ppb increase in lag 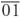 daily O_3_ levels were 0.25% (0.12 to 0.38%), 0.18% (0.07 to 0.30%), 0.14% (0.03 to 0.26%), and 0.04% (–0.07 to 0.16%) for single-day lags of 0, 1, 2, and 3 days, respectively. The community-specific maximum likelihood estimates under the linear approach displayed no association with the communities’ long-term O_3_ concentrations over the study period, as tested by correlation and weighted second-stage regression.

Our results show that daily increases in ambient O_3_ were significantly associated with daily increases in the number of deaths, on average, across the 98 U.S. communities for the idealized policy scenarios under which every community meets current O_3_ regulatory standards and guidelines (California Environmental Protection Agency 2005; Canadian Council of Ministers of the Environment 2000; European Commission 2002; U.S. EPA 1997; WHO 2000) for every day of the study period, 1987–2000 (Table 1). For example, the percentage increase in all-cause mortality associated with a 10-ppb increase in lag 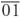 O_3_ levels was 0.30% (0.15–0.45%) when we used a data set including only days with a daily 8-hr maximum O_3_ concentration lower than U.S. O_3_ regulations. We also found that daily increases in ambient O_3_ exposure are linked to premature mortality under compliance with other O_3_ regulations, including some more stringent than the U.S. standards. In summary, these results indicate that current regulations, even California’s new, more stringent standards, are not sufficiently low to provide complete protection against the risk of premature mortality from O_3_.

**Figure 2:**
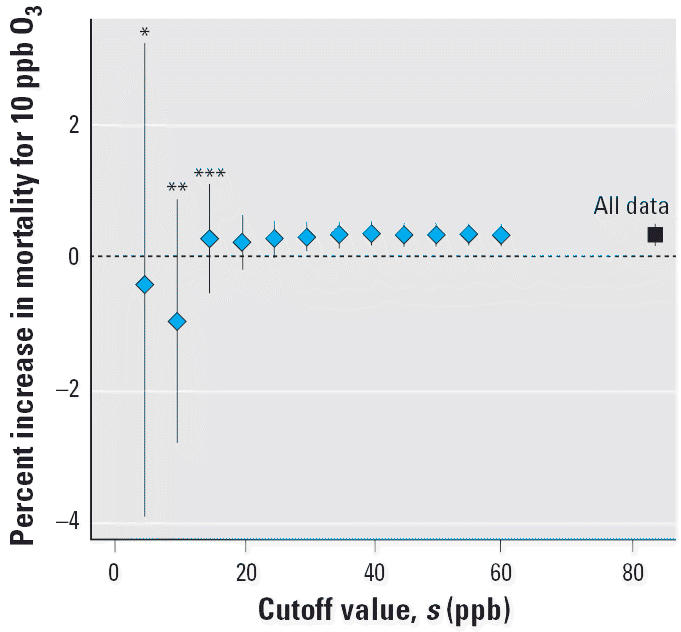
Percentage increase in daily nonaccidental mortality per 10-ppb increase in lag 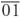 O_3_ obtained by using the subset approach. Diamonds denote the point estimates, and vertical lines represent the 95% posterior intervals. Each estimate is obtained by including in the analysis only days with 24-hr average lag 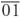 O_3_ levels below the *s* value specified on the x-axis. Not all communities had sufficient data for analysis at all *s* values: *25 communities; **74 communities; ***92 communities. All other estimates used 98 communities. The estimate at the far right marked by a square uses all data.

Daily changes in ambient O_3_ were significantly associated with daily changes in the number of deaths, on average, across the 98 U.S. communities, even when we used data that include only days with lag 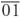 average O_3_ levels < 15 ppb. Figure 2 shows the estimated percentage increase in mortality for a 10-ppb increase in the lag 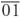 O_3_ level for different values of *s*. National relative rate estimates for *s* values ranging from 35 to 60 ppb are similar to the ones obtained by using all data. The 95% posterior interval increases as *s* is lowered because of the decreasing sample size. For example, at an *s* value of 40 ppb, 30% of days are excluded from analysis, on average, across the 98 communities. At an *s* of 20 ppb, 73% of days are excluded. The estimates decline and lose significance only when *s* is equal to very low concentrations (≤ 10 ppb). Therefore, the subset approach suggests that a “safe” O_3_ level would be lower than approximately 10 ppb, for the lag 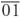 daily O_3_ level, which is roughly 15–19 ppb for the maximum 8-hr average. However, relative rate estimates for *s* ≤ 10 ppb have large statistical uncertainty because of the very small number of days with O_3_ concentrations so low. In fact, 73 communities were excluded entirely at an *s* of 5 ppb because of insufficient data.

**Figure 3:**
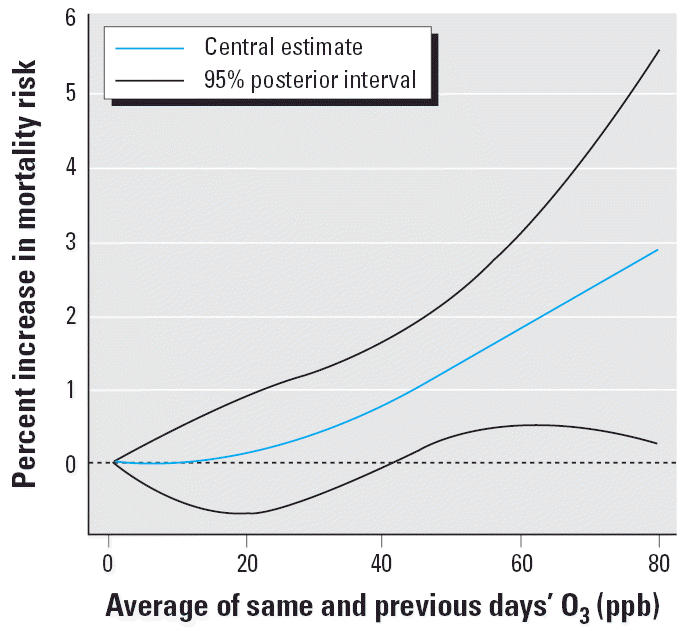
Exposure–response curve for O_3_ and mortality using the spline approach: percentage increase in daily nonaccidental mortality at various O_3_ concentrations.

Results from the threshold and spline approaches are consistent with those from the subset approach and provide evidence that a “safe” O_3_ level can only exist at very low concentrations. We found that the model fit under the threshold approach for values of *h* from 5 to 60 ppb never provides more than a nominal improvement (< 1% difference in the 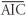) over the model fit under the linear approach (analogous to the threshold approach with *h* = 0) for the national average and each individual community. In other words, a model that allows for a “safe” O_3_ level fit the data only marginally better than a model that assumes any level of O_3_ pollution, even low concentrations, can be associated with mortality. The spline approach indicates that the national average exposure–response curve obtained using natural cubic splines is near horizontal, indicating the lack of evidence for an association, only at the very low concentrations (from 0 to ~10 ppb) and then becomes approximately linear at higher concentrations (Figure 3).

## Discussion

In summary, our nationwide study provides strong and consistent evidence that daily changes in ambient O_3_ exposure are linked to premature mortality, even at very low pollution levels, including an idealized scenario of complete adherence to current O_3_ regulations. We also found robust evidence of this relationship between O_3_ exposure and mortality when we used data that included only O_3_ levels nearing background concentrations, which typically range from 10 to 25 ppb (Fiore et al. 2003, 2004). Therefore, any anthropogenic contribution to ambient O_3_, however slight, still presents an increased risk for premature mortality.

Results from this multisite national study are consistent with single-site time-series studies that found no evidence of a “safe” O_3_ leve at concentrations higher than background levels. Consistent with the results obtained under our spline approach, Kim et al. (2004) found that a spline model indicated a threshold around 20–30 ppb for the daily 1-hr maximum, which is approximately equal to 8–12 ppb for the 24-hr average, using 5 years of data for Seoul, Korea. Hoek et al. (1997) found that relative risk estimates of mortality associated with daily changes in O_3_ were robust to exclusion of days with a 24-hr average ≥ 40 μg/m^3^ (about 20 ppb) in a study of Rotterdam, the Netherlands, and concluded that should a threshold exist, it may be at a low concentration. Adverse health responses such as decreases in pulmonary function, alterations in the respiratory tract, and declines in lung function have been observed at O_3_ levels close to background concentrations (Chan and Wu 2005; WHO 2000). O_3_ levels below U.S. EPA regulations have been associated with increased frequency of respiratory symptoms in children with asthma (Gent et al. 2003).

Pollution levels below air quality regulatory standards should not be misinterpreted as safe for human health. For instance, the San Joaquin Valley Air Pollution Control District refers to the standards as the “highest level of O_3_ that can be present without adverse health effects” (San Joaquin Valley Air Pollution Control District 2006). However, decision makers and the public should distinguish between the complete absence of harm and a lessened or acceptable risk. In fact, the interpretation of an “adequate margin of safety” and what is a “safe” level could depend on the individual, because people may differ in their susceptibility to air pollutants, and could depend on the evolving knowledge about the health impacts of air pollution at low levels (American Thoracic Society 2000). This research shows that any reduction in ambient O_3_ levels, such as through transportation planning in urban areas, should yield important benefits to public health, even in areas that meet current regulatory standards. Persons may be adversely affected by O_3_ pollution, even at very low levels including days that meet current regulatory requirements.

## Figures and Tables

**Appendix:** List of 98 U.S. urban communities.

Akron, Ohio	Des Moines, Iowa	Lincoln, Nebraska	Riverside, California
Albuquerque, New Mexico	Detroit, Michigan	Little Rock, Arkansas	Rochester, New York
Arlington, Virginia	District of Columbia	Louisville, Kentucky	Sacramento, California
Atlanta, Georgia	El Paso, Texas	Los Angeles, California	Salt Lake City, Utah
Austin, Texas	Evansville, Indiana	Madison, Wisconsin	San Antonio, Texas
Bakersfield, California	Fort Wayne, Indiana	Memphis, Tennessee	San Bernardino, California
Baltimore, Maryland	Fresno, California	Miami, Florida	San Diego, California
Baton Rouge, Louisiana	Grand Rapids, Michigan	Milwaukee, Wisconsin	San Jose, California
Biddeford, Maine	Greensboro, North Carolina	Mobile, Alabama	Santa Ana/Anaheim, California
Birmingham, Alabama	Honolulu, Hawaii	Modesto, California	
Boston, Massachusetts	Houston, Texas	Muskegon, Michigan	Seattle, Washington
Buffalo, New York	Huntsville, Alabama	Nashville, Tennessee	Shreveport, Louisiana
Cedar Rapids, Iowa	Indianapolis, Indiana	New Orleans, Louisiana	Spokane, Washington
Charlotte, North Carolina	Jackson, Mississippi	New York, New York	St. Louis, Missouri
Chicago, Illinois	Jacksonville, Florida	Newark, New Jersey	St. Petersburg, Florida
Cincinnati, Ohio	Jersey City, New Jersey	Oakland, California	Stockton, California
Cleveland, Ohio	Johnstown, Pennsylvania	Oklahoma City, Oklahoma	Syracuse, New York
Colorado Springs, Colorado	Kansas City, Kansas	Omaha, Nebraska	Tacoma, Washington
Columbus, Georgia	Kansas City, Missouri	Orlando, Florida	Tampa, Florida
Columbus, Ohio	Kingston, New York	Philadelphia, Pennsylvania	Toledo, Ohio
Corpus Christi, Texas	Knoxville, Tennessee	Phoenix, Arizona	Tucson, Arizona
Coventry, Rhode Island	Lafayette, Louisiana	Pittsburgh, Pennsylvania	Tulsa, Oklahoma
Dallas/Fort Worth, Texas	Lake Charles, Louisiana	Portland, Oregon	Wichita, Kansas
Dayton, Ohio	Las Vegas, Nevada	Providence, Rhode Island	Worcester, Massachusetts
Denver, Colorado	Lexington, Kentucky	Raleigh, North Carolina	

Descriptive statistics for each community are given in iHAPSS (2006).

**Table 1:** National effect estimates (95% posterior interval) under the scenario that a specific regulation or guideline is met every day in each community.

Organization/government	Regulation/guideline	Increase in mortality for 10-ppb increase in lag 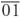 O_3_ (%)
U.S. EPA	84 ppb daily 8-hr maximum	0.30 (0.15–0.45)
WHO (guideline)	120 μg/m^3^ (~ 61 ppb) daily 8-hr maximum	0.25 (0.06–0.43)
European Commission (target value for 2010)	120 μg/m^3^ (~ 61 ppb) daily 8-hr maximum	0.25 (0.06–0.43)
Canada (to be achieved by 2010)	65 ppb daily 8-hr maximum	0.28 (0.11–0.45)
California	70 ppb daily 8-hr maximum	0.30 (0.14–0.46)
	90 ppb daily 1-hr maximum	0.29 (0.14–0.44)
	Both of California’s above standards	0.31 (0.14–0.47)
All standards	All of the above standards and guidelines	0.24 (0.06–0.42)
All days of data^a^	NA	0.32 (0.17–0.46)

NA, not applicable.

a Considered regardless of whether they meet a standard or guideline.
